# What is the risk of acquiring bacteria from prior intensive care unit bed occupants?

**DOI:** 10.1186/s13054-017-1652-y

**Published:** 2017-03-22

**Authors:** Vincenzo Russotto, Andrea Cortegiani, Santi Maurizio Raineri, Pasquale Iozzo, Cesare Gregoretti, Antonino Giarratano

**Affiliations:** 1 Department of Biopathology and Medical Biotechnologies (DIBIMED), Section of Anaesthesia, Analgesia, Intensive Care and Emergency, Policlinico Paolo Giaccone, University of Palermo, Via del Vespro 129, 90127 Palermo, Italy; 2 Intensive Care Unit, Policlinico Paolo Giaccone, University of Palermo, Via del Vespro 129, 90127 Palermo, Italy

**Keywords:** Bacterial contamination, Multi-drug resistant bacteria, Infection, Sepsis

Contamination of inanimate surfaces and equipment may play a role in cross-transmission of bacteria in the intensive care unit (ICU), despite current standards of terminal cleaning [1–3]. This is particularly relevant due to the high prevalence of multidrug-resistant (MDR) colonization of critically ill patients and the virulence and invasiveness of bacteria, which frequently present multidrug resistance to antimicrobials (e.g. *Staphylococcus aureus*, *Acinetobacter baumanii*, *Klebsiella pneumoniae*). Moreover, colonization has been identified as a risk factor for subsequent infections in critically ill patients [4, 5].

We searched Medline, Scopus, and CINHAL databases for either prospective or retrospective studies reporting data on ICU-acquired bacterial species and carriage status of the same species by the prior bed occupant. An exclusion criterion was the non-ICU setting due to differences in terminal cleaning procedures and the hospital environment of the patients. We applied no language restrictions and did not consider gray literature. We performed a random-effect meta-analysis (Mantel-Haenszel method) of included studies and calculated the odds ratio (OR) for acquiring specific microorganisms as well as from the pooled data (Fig. 1). We assessed the heterogeneity among studies by the *I* ^2^ statistic.

**Fig. 1:**
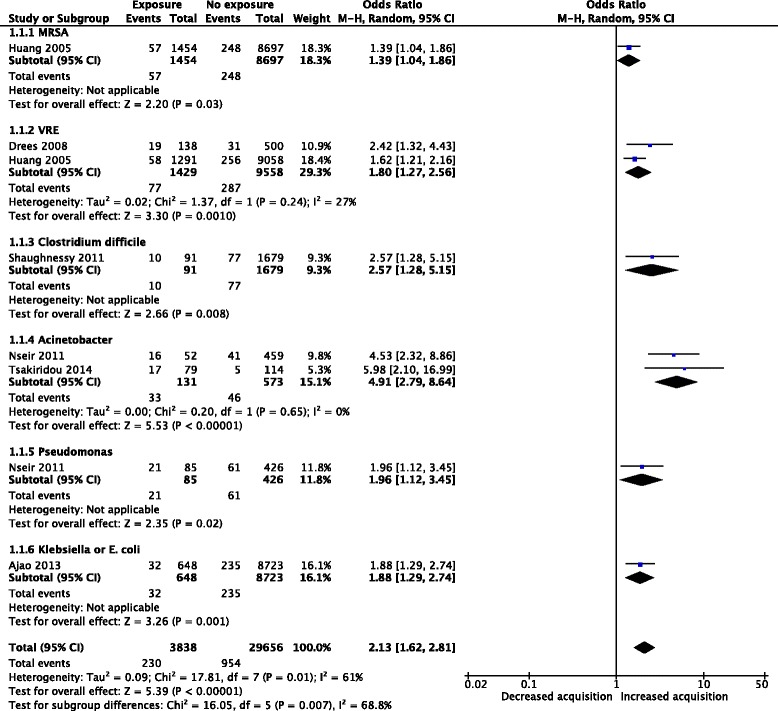
Forest plot of studies evaluating the risk of acquiring bacteria from prior ICU bed occupants. *CI* confidence interval, *M-H* Mantel-Haenszel, *MRSA* methicillin-resistant *Staphylococcus aureus*, *VRE* vancomycin-resistant enterococci

The full search strategy can be found as Additional file 1. Our search strategy found a total of 2264 articles from inception to 1 January 2017 (1247 Medline, 654 Scopus, 363 CINHAL). Two authors (VR and AC) independently performed the search and extracted the data. In case of disagreement, it was solved by consensus with another author (SMR). We used Review Manager 5.3.

We selected six studies for inclusion (two prospective cohort study, one post-hoc analysis of a prospective interventional cross-over study, and three retrospective studies) for a total of 33,494 patients. The list of included studies and references is available as Additional file 2. Among 3838 patients admitted to ICU beds with prior infected/colonized occupants, 230 acquired bacteria compared to 954 among 29,656 in the control group. The overall pooled OR of acquiring a bacterial pathogen from prior ICU bed occupants was 2.13 (95% confidence interval 1.62–2.81). We observed a substantial heterogeneity among included studies (*I* ^2^ = 61%, *P* = 0.01). Figure 1 shows the forest plot of meta-analysis of studies with subgroup analyses according to microorganisms and pooled data.

Patients admitted to ICU beds of prior occupants who were carrying bacterial pathogens should be considered at increased risk of ICU-acquisition compared to other ICU patients. It should be highlighted that these results refer to acquisition of bacteria and not to infection. However, it may be argued that acquiring bacteria may lead to colonization and nosocomial infections during the ICU stay due to disruption of natural barriers for invasive procedures (e.g., insertion of central venous catheters, arterial lines), use of broad-spectrum antibiotics, and impaired immunological function. A possible explanation for these results is that bacteria frequently encountered in the ICU, especially MDR, may have the ability to survive standard terminal cleaning procedures in the environment close to the patients after discharge. Further research should evaluate the role of bacteria acquisition from prior ICU bed occupants in terms of patient-related outcomes and the impact of new strategies for terminal cleaning to reduce hospital-acquired infection.

## Additional files

Additional file 1: Search strategy. Search strategy adopted for the systematic review. (PDF 34 kb)

Additional file 2: List of included studies. Table reporting the studies included in the meta-analysis. (PDF 62 kb)

## Abbreviations

CI: Confidence interval
HAI: Hospital-acquired infections
ICU: Intensive care unit
MDR: Multidrug resistant
OR: Odds ratio
