# Supplementary material for: What is the risk of acquiring bacteria from prior intensive care unit bed occupants?
Source: Crit Care. 2017 Mar 22;21:55. doi: 10.1186/s13054-017-1652-y (PMC5361701; doi:10.1186/s13054-017-1652-y)
Supplement: Supplementary file 1 — Search strategy. Search strategy adopted for the systematic review. (PDF 34 kb) [file 13054_2017_1652_MOESM1_ESM.pdf]

Search strategy

(prior room OR occupancy OR prior room occupancy OR intensive care unit room OR bed OR intensive care unit bed OR intensive care bed) AND (transmission OR acquisition OR infection transmission OR infection acquisition OR contamination) AND (acinetobacter OR escherichia AND coli OR klebsiella OR pseudomonas OR enterobacter OR proteus OR serratia OR enterococcus OR clostridium AND difficile OR staphylococcus AND aureus OR methicillin resistant AND staphylococcus AND aureus OR vancomycin resistant AND enterococcus OR gram-positive OR gram negative OR multidrug AND resistant AND bacteria OR bacteria OR microorganisms)
