# Supplementary material for: What is the risk of acquiring bacteria from prior intensive care unit bed occupants?
Source: Crit Care. 2017 Mar 22;21:55. doi: 10.1186/s13054-017-1652-y (PMC5361701; doi:10.1186/s13054-017-1652-y)
Supplement: Supplementary file 2 — List of included studies. Table reporting the studies included in the meta-analysis. (PDF 62 kb) [file 13054_2017_1652_MOESM2_ESM.pdf]

Additional file 2 showing the list of included studies.

STUDY ID	TITLE	REFERENCE
Ajao 2013	Risk of acquiring extended spectrum β-lactamase-producing Klebsiella species and Escherichia coli from prior room occupants in the intensive care unit	Infect Control Hosp Epidemiol 2013;34:453e458
Drees 2008	Prior environmental contamination increases the risk of acquisition of vancomycin resistant enterococci.	Clin Infect Dis 2008;46:678e685.
Huang 2006	Risk of acquiring antibiotic-resistant bacteria from prior room occupants	Arch Intern Med. 2006;166(18):1945-1951.
Nseir 2011	Risk of acquiring multidrug-resistant Gram-negative bacilli from prior room occupants in the intensive care unit	Clin Microbiol Infect 2011; 17: 1201–1208
Shaughnessy 2011	Evaluation of hospital room assignment and acquisition of Clostridium difficile infection	Infect Control Hosp Epidemiol 2011;32:201e206
Tsakiridou 2014	Acinetobacter baumannii infection in prior ICU bed occupants is an independent risk factor for subsequent cases of ventilator-associated pneumonia.	Biomed Res Int. 2014;2014:193516.
